# Diagnostic value of different anti-citrullinated peptides antibodies in rheumatoid arthritis

**DOI:** 10.1186/1479-5876-9-S2-P51

**Published:** 2011-11-23

**Authors:** Estíbaliz Ruiz-Ortiz de Arrizabaleta, Dolors Grados-Cánovas, Aina Teniente-Serra, Virginia García-López, Bibiana Quirant-Sánchez, Susana Holgado, Alejandro Olivé, Ricardo Pujol-Borrell, Eva Martínez-Cáceres

**Affiliations:** 1 Immunology Laboratory, Banc de Sang I Teixits, Hospital Universitari Germans Trias i Pujol (HUGTP), Badalona, Spain; 2 Dept. of Cell Biology, Physiology and Immunology, Faculty of Medicine, Universitat Autònoma Barcelona, Spain; 3 Rheumatology Division, HUGTP, Badalona, Spain; 4 Immunology Division, Hospital Universitari Vall d’Hebron, Barcelona, Spain

## Introduction

Assays that detect anti-citrullinated peptides antibodies (ACPA) are considered to be more specific than rheumatoid factor in the diagnosis of rheumatoid arthritis (RA). Several tests have been developed using different antigens: first, second and third-generation cyclic-ACPA (CCP1, CCP2, CCP3) and modified citrullinated vimentin (MCV).

## Aim

To investigate anti-CCP3 in a group of patients positive citrullinated vimentin antibodies (MCVA).

## Patients and methods

A total of 259 patients positive for IgG MCVA+ attending the outpatient rheumatology clinic of a single general hospital (HUGTiP) were tested for anti-CCP3 (by ELISA). From the total, 182 (70.3%) of them had a rheumatic disease (RD): RA in 121 (66.5%) and other RDs in 61 (33.5%). 77 (29.7%) patients with other conditions positive for MCVA were also tested for anti-CCP3.

## Results

From the 121 RA patients, 106 (87.6%) were positive for anti-CCP3. In contrast, only 15 (24.6%) of the 61 patients without RA and only 4 (5.2%) of the 77 MCVA+ patients with no RD associated were CCP3 positive. Interestingly, within the group no RA, of the 13 patients with anti-CCP3 values >60 U/ml, 6 (46.2%) were cases of palindromic rheumatism and two of them had developed RA. Specificity of anti-CCP3 for RA as compared to other RDs was 76.7%, that raised to 86.9% when comparing RA versus to non-RA (with or without another RDs). Positive and negative predictive values (PPV, NPV) of anti-CCP3 for RA were 85.5% and 88.8%, respectively whereas PPV for anti-MCV was 39.1%.

## Conclusions

Anti-CCP3 antibodies show a higher specificity for RA when compared to MCVA with a better PPV, a crucial feature for a test in use for clinical practice.

